# Four-Year Durability of Initial Combination Therapy with Sitagliptin and Metformin in Patients with Type 2 Diabetes in Clinical Practice; COSMIC Study

**DOI:** 10.1371/journal.pone.0129477

**Published:** 2015-06-12

**Authors:** Eu Jeong Ku, Kyong Yeon Jung, Yoon Ji Kim, Kyoung Min Kim, Jae Hoon Moon, Sung Hee Choi, Young Min Cho, Kyong Soo Park, Hak Chul Jang, Soo Lim, Bo Ahrén

**Affiliations:** 1 Department of Internal Medicine, Seoul National University College of Medicine, Seoul, Republic of Korea; 2 Department of Internal Medicine, Seoul National University Bundang Hospital, Seongnam, Republic of Korea; 3 Department of Internal Medicine, Chungbuk National University Hospital, Cheongju, Republic of Korea; 4 Department of Clinical Sciences, Lund University, Lund, Sweden; Università “Magna Graecia” di Catanzaro, ITALY

## Abstract

**Objectives:**

We investigated the efficacy of initial combination therapy with sitagliptin and metformin in patients with type 2 diabetes for 4 years in clinical practice.

**Methods:**

Between 2009 and 2010, we reviewed 1,178 patients with type 2 diabetes (HbA_1c _≥7.5% or 58 mmol/mol) prescribed initial combination therapy with sitagliptin and metformin. After excluding 288 patients without a second follow-up, 890 individuals (age, 58.0 ± 12.5 years; BMI, 25.4 ± 3.5 kg/m^2^; HbA_1c_, 8.6 ± 1.1%) were followed up with every 3–6 months for 4 years. Homeostasis model assessments for insulin resistance and β-cell function (HOMA-β) were recorded at baseline. The response criterion was HbA_1c_ reduction by ≥0.8% from baseline or attainment of the target HbA_1c_ (≤7.0% or 53 mmol/mol). At the end of every year of treatment, changes in HbA_1c_ from the baseline were assessed.

**Results:**

After 1 year, 72.2% of patients with initial combination therapy had responded, defined as HbA_1c_ reduction ≥0.8% or attainment of the target HbA_1c _≤7.0%. After 4 years, 35.4% of the patients still showed a response, with an HbA_1c_ level of 7.0 ± 0.9%. A high HbA_1c_ level at baseline was the most significant independent predictor of the long-term response (*P*<0.001). In addition, low HOMA-β was a significant predictor of a greater reduction in HbA_1c_. This treatment was generally well tolerated over the 4-year follow-up period, without any serious adverse events.

**Conclusions:**

This real-world follow-up study shows a persistent glucose-reducing effect of initial combination therapy with sitagliptin and metformin for up to 4 years.

## Introduction

Sitagliptin is a highly selective and orally active dipeptidyl peptidase-4 (DPP-4) inhibitor [1], which was approved by the US Food and Drug Administration in 2006. Previous randomized clinical studies have shown that sitagliptin monotherapy reduced the glycosylated hemoglobin (HbA_1c_) level by 0.4–0.6% [2–4], and sitagliptin combination therapy with metformin or thiazolidinedione decreased HbA_1c_ by 0.4–1.4% over 18–52 weeks of treatment in patients with type 2 diabetes (T2D) [3,5,6]. More specifically, the clinical trials with sitagliptin and metformin as initial combination therapy have shown an average reduction of HbA_1c_ by ≥0.8% [6,7].

Although more than 4 years have passed since sitagliptin first became available in clinical practice, previous studies have only reported the efficacy and safety of sitagliptin with or without metformin over a 2-year time frame [8–12]. In recent years, a combination of a DPP-4 inhibitor and metformin has commonly been used, demonstrating the additive effects resulting from complementary mechanisms of action [5,13]. However, there have been few studies of the long-term durability and safety of combination therapy, particularly in the real-world clinical setting.

We investigated the long-term durability and safety of initial combination therapy with sitagliptin and metformin in patients with T2D in clinical practice. We also evaluated the predictive markers for therapeutic efficacy of the coadministration of sitagliptin and metformin.

## Patients and Methods

### Subjects

In this retrospective cohort study, we reviewed all drug-naïve patients with T2D who underwent an initial combination treatment of sitagliptin and metformin in the diabetes clinic at Seoul National University Bundang Hospital (SNUBH), Seongnam, Korea, from 2009 to 2010. The inclusion criteria were 1) baseline HbA_1c_ of 7.5% or higher, 2) drug-naïve and no current antidiabetic agent within 6 months before enrollment, and 3) no other drug taken that could influence glucose metabolism such as steroids, alternative medicine containing steroid, immunosuppressive agents, and appetite suppressants. The exclusion criteria were 1) diagnosis of type 1 diabetes mellitus confirmed by the presence of glutamic acid decarboxylase antibody, 2) gestational diabetes, 3) diabetes due to secondary causes, and 4) cancer patients on active anticancer treatment. Of the 3,783 patients screened, 1,178 fulfilled the inclusion and exclusion criteria. Of these, 288 patients without a second follow-up were excluded. A total of 890 patients were treated for more than 90 days with initial combination therapy with sitagliptin and metformin and were followed up more than twice; these patients formed the study cohort.

This study was approved by the Institutional Review Board of Seoul National University Bundang Hospital (No. B-1311-228-110). All procedures followed were in accordance with the ethical standards of the responsible committee on human experimentation (institutional and national) and with the Declaration of Helsinki 1975, as revised in 2008. The need for informed consent was waived by the Institutional Review Board of Seoul National University Bundang Hospital.

### Study design

We provided pertinent diabetes education, including a therapeutic lifestyle change program, to standardize every patient’s education level. All patients were regularly followed up at intervals of 3–6 months. At every clinic visit, the patients’ medical history and laboratory values were reviewed. For all subjects, drug compliance was also assessed by recording the number of pills that had not yet been taken at every visit during follow-up. The criterion for poor compliance was that <80% of the prescribed pills had been taken. The physicians were able to change the antidiabetic agents at their discretion.

**Fig 1:**
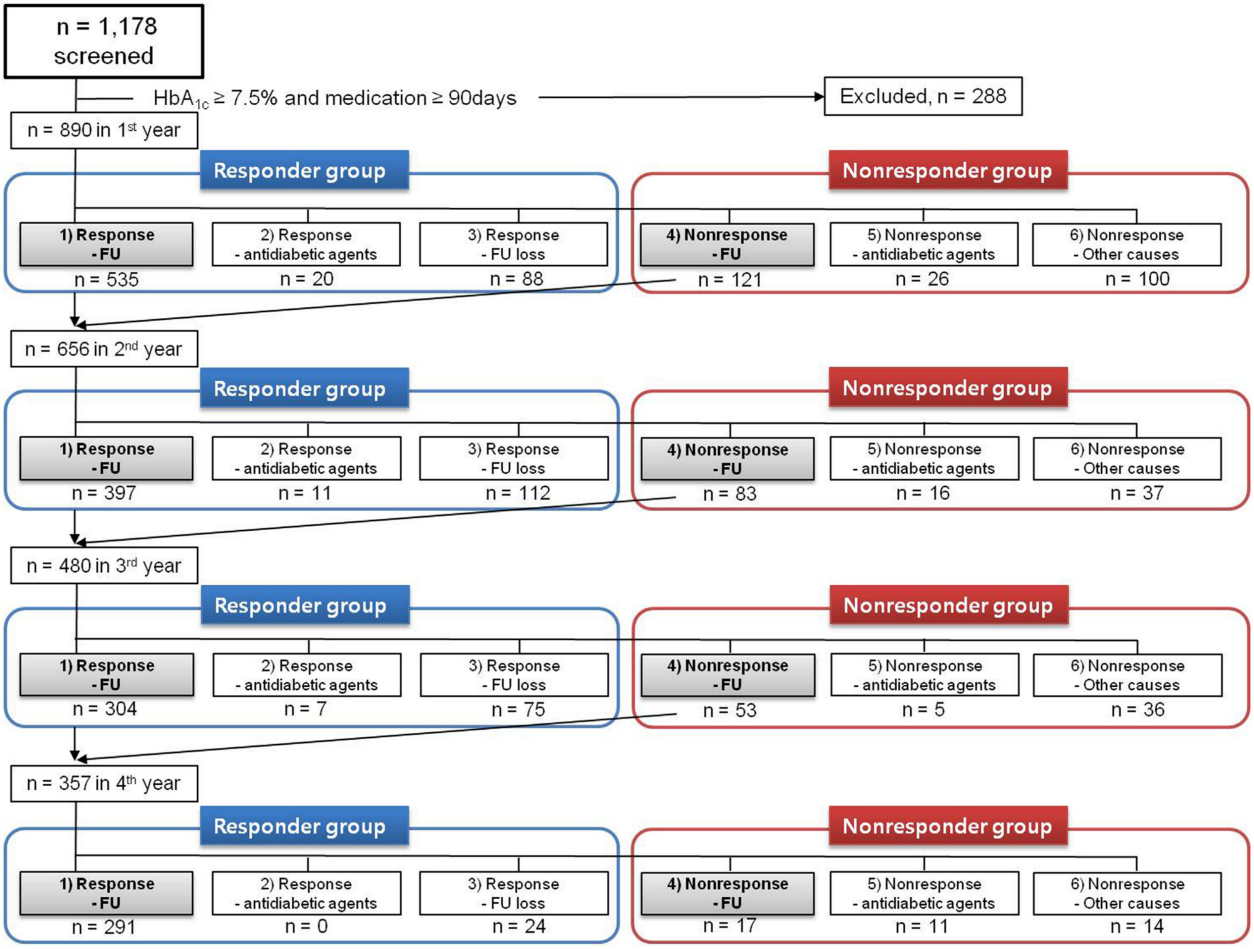
Study disposition. FU; follow-up.

During the follow-up period, patients were assigned to either a responder or a nonresponder group based on their response, with a response defined as a HbA_1c_ reduction ≥0.8% from the baseline or attainment of the target HbA_1c_ (≤7.0%) (Fig 1).

In the responder group, there were three subgroups: 1) Response-FU; patients with a response who continued to be followed up, 2) Response-antidiabetic agents; patients with a response (HbA_1c_ reduction ≥0.8% from the baseline) but for whom one or more additional antidiabetic agents were either added or used to replace sitagliptin for achieving the target HbA_1c_ (≤7.0%), and 3) Response-FU loss; patients with a response but who were lost to follow-up. The most common reasons for loss to follow-up were moving away, migration to another country, feeling that the medication had a low efficacy, thinking that no further treatment was necessary, or no specific reason.

In the nonresponder group, there were three subgroups: 4) Nonresponse-FU; patients without a response but who continued to be followed up, 5) Nonresponse-antidiabetic agents; patients without a response for whom one or more additional antidiabetic agents were either added or used to replace sitagliptin, 6) Nonresponse-other causes; patients without a response who were lost to follow-up; patients without a response excluded for other reasons such as the use of other medications such as systemic steroids, anticancer drugs, or weight-control drugs, malignancy, chronic wasting disease such as tuberculosis or malabsorption diseases, or organ transplantation; patients without a response because of poor compliance (<80%) with the prescribed sitagliptin and metformin medication regimen.

To assess long-term durability, the patients who belonged to the Response-FU and Nonresponse-FU subgroups (subgroups 1 and 4) continued onto the next year and were categorized into one of the six subgroups using the same indications every year for 4 years. The response rate was evaluated at the end of each year and was calculated as the number of patients in the responder group (i.e. subgroups 1–3) divided by all patients enrolled in the study for that year (i.e. subgroups 1–6).

### Primary efficacy assessments

The primary efficacy outcome was the change in HbA_1c_ from the baseline to 48 months. HbA_1c_ levels were also assessed at the end of every year of treatment to assess the rate of response, which was defined as HbA_1c_ reduction ≥0.8% from the baseline or attainment of the target HbA_1c_ (≤7.0%). The long-term responders were defined as subjects who continued to respond to the treatment over the entire 4-year study period. The early nonresponders were the subjects who did not respond within 1 year following treatment.

### Anthropometric and biochemical parameters at baseline and during follow-up

At each visit, BMI was calculated and blood pressure was measured. Smoking status was divided into two categories: ever smokers and never smokers. Alcohol intake in grams of alcohol per week was categorized into two intake categories: moderate (<200 g/week) and heavy (≥200 g/week). Exercise habit was divided into two categories: none or irregular and regular exercise (≥3 times per week).

After 12 h of overnight fasting, venous blood samples were taken for biochemical assays at the baseline and at intervals of 3–6 months. Plasma glucose concentration was measured using the glucose oxidase method (Hitachi 747 chemistry analyzer; Hitachi, Tokyo, Japan). HbA_1c_ was measured with a Bio-Rad Variant II Turbo HPLC analyzer (Bio-Rad, Hercules, CA, USA) in the SNUBH, a National Glycohemoglobin Standardization Program (NGSP) level II-certified laboratory. Total cholesterol, triglycerides, low-density lipoprotein cholesterol (LDL-C), high-density lipoprotein cholesterol (HDL-C), aspartate aminotransferase (AST), and alanine aminotransferase (ALT) were measured with a Hitachi 747 chemistry analyzer (Hitachi). Estimated glomerular filtration rate (eGFR) was calculated by the Modification of Diet in Renal Disease equation [14]. Fasting insulin and C-peptide levels were measured by radioimmunoassay (Millipore, St. Louis, MO, USA) at the first visit and at the physician’s discretion. The homeostasis model assessment of insulin resistance (HOMA-IR) and β-cell function (HOMA-β) were calculated [15].

### Safety assessments

For the safety evaluation, vital signs, physical examinations, medical history, laboratory values, and adverse clinical experiences were assessed at every clinic visit. Hypoglycemia was defined as a blood glucose concentration <70mg/dl (3.9 mmol/l). Adverse clinical experiences included any clinical events that could be related to sitagliptin or metformin therapy. The investigators assessed the relationship of all adverse clinical experiences to the study drugs.

### Statistical analyses

Statistical analyses were performed using SPSS for Windows (version 20.0, IBM Corp., Armonk, NY, USA). All data were shown as means ± standard deviations. The baseline characteristics were compared with Student’s *t*-test. To compare the continuous variables between baseline and posttreatment, a paired *t*-test was used. Correlations between variables were analyzed using Pearson’s correlation and Fisher’s exact test. We tested the independent associations of β-cell function and insulin resistance, assessed using the tertiles of HOMA-β and HOMA-IR, with changes in HbA_1c_ levels. Multiple regression analyses were conducted to evaluate the predictive factors for the long-term durable response to combination treatment with sitagliptin and metformin; age, sex, systolic blood pressure, BMI, duration of diabetes before treatment, triglyceride, HDL-C, ALT, eGFR, HOMA-β and HOMA-IR were managed as continuous variables, and family history of diabetes, alcohol consumption, smoking history, and exercise habit as categorical variables. In model 1, age, sex, systolic blood pressure, BMI, duration of diabetes, family history of diabetes, alcohol consumption, smoking habit, and exercise were included. In model 2, biochemical factors were added to model 1. In model 3, HOMA-β and HOMA-IR were added. Finally, baseline HbA_1c_ level was added to model 4. Depending on the baseline HbA_1c_ levels, subgroup analysis was performed to assess the predictive factors for treatment response [baseline HbA_1c_<8.5% and ≥8.5%]. A *P*<0.05 was considered significant.

## Results

### Baseline characteristics

The baseline demographic and laboratory characteristics of 890 patients are summarized in Table 1. The mean age was 58.0 ± 12.5 years and 61.9% (n = 551) were male. The mean duration of diabetes was 3.4 ± 2.7 years. The HbA_1c_ level at the baseline was 8.6 ± 1.1%. The baseline clinical characteristics were the same for the excluded patients (n = 288) and the study participants (n = 890) (data not shown).

**Table 1:** Baseline demographic and laboratory data for study subjects.

Variable	All patients treated (n = 890)
Age, years	58.0 ± 12.5
Male, n (%)	551 (61.9)
Systolic blood pressure, mmHg	126.0 ± 15.5
Diastolic blood pressure, mmHg	75.4 ± 10.9
Body weight, kg	68.0 ± 12.4
Body mass index, kg/m2	25.4 ± 3.5
Duration of diabetes, years	3.4 ± 2.7
Family history of diabetes, n (%)	191(21.5)
Comorbid disease, n (%)	
Coronary heart disease	147 (16.5)
Cerebrovascular disease	114 (12.8)
Current medication, n (%)	
Statin	474 (53.3)
ACEi or ARB	398 (44.7)
β-blocker	104 (11.7)
Alcohol consumption, n (%)	
Moderate	851 (95.6)
Heavy	39 (4.4)
Smoking status, n (%)	
Never smoker	692 (77.8)
Ever smoker	198 (22.2)
Regular exercise, n (%)	
None or irregular	627 (70.4)
Regular	263 (19.5)
HbA1c, % (mmol/mol)	8.6 ± 1.1 (70.0 ± 12.3)
Fasting plasma glucose, mg/dL	163.3 ± 48.7
2-PP, mg/dL	264.6 ± 82.4
Fasting insulin, μIU/mL	13.9 ± 6.5
C-peptide, ng/mL	2.4 ± 1.2
HOMA-IR	5.8 ± 3.3
HOMA-β, %	59.8 ± 40.5
Total cholesterol, mg/dL	182.3 ± 39.7
Triglyceride, mg/dL	184.0 ± 139.0
HDL cholesterol, mg/dL	46.5 ± 10.8
LDL cholesterol, mg/dL	101.7 ± 33.7
Aspartate aminotransferase, IU/L	25.5± 13.3
Alanine aminotransferase, IU/L	31.4± 20.7
eGFR, ml/min/1.73 m2	82.3± 20.4

Data are expressed as mean ± standard deviation or number (percentage). ACEi, angiotensin converting enzyme inhibitor; ARB, angiotensin receptor blocker; HbA_1c_, hemoglobin A1c; 2-PP, postprandial 2 h glucose; HOMA-IR and HOMA-β, homeostasis model assessment of insulin resistance and β-cell function (n = 812); eGFR, estimated glomerular filtration rate calculated by the Modification of Diet in Renal Disease equation.

### Patient follow-up rates

The numbers of patients in the responder and nonresponder groups in each year are shown in Fig 1. Of the 890 study subjects at the baseline, 656 patients who belonged to subgroups 1 and 4 progressed to the second year, resulting in a follow-up rate of 73.7% at the end of the first year. The follow-up rates at the end of the second, third, and fourth years were 73.2%, 74.4%, and 86.3%, respectively. As a percentage of the baseline cohort, the follow-up rates were 53.9%, 40.1% and 34.6%, respectively.

### Efficacy outcomes

Of the 890 patients, 643 patients in subgroups 1–3 were categorized into the responder group, resulting in a response rate of 72.2% during the first year of medication. From the second year to the fourth year, the response rate increased from 79.3% to 88.2% (*P*<0.01). At the end of the fourth year, absolutely 35.4% (315/890) of the patients were remained as responders. Initial combination therapy with sitagliptin and metformin showed a significant reduction in the HbA_1c_ level; 1.5% (from 8.6 ± 1.1% to 7.1 ± 1.0%) in the first year, 1.5% (7.1 ± 1.0%) in the second year, 1.7% (6.9 ± 0.8%) in the third year, and 1.6% (7.0 ± 0.9%) in the fourth year (all *P*<0.001 vs. baseline HbA_1c_).

**Fig 2:**
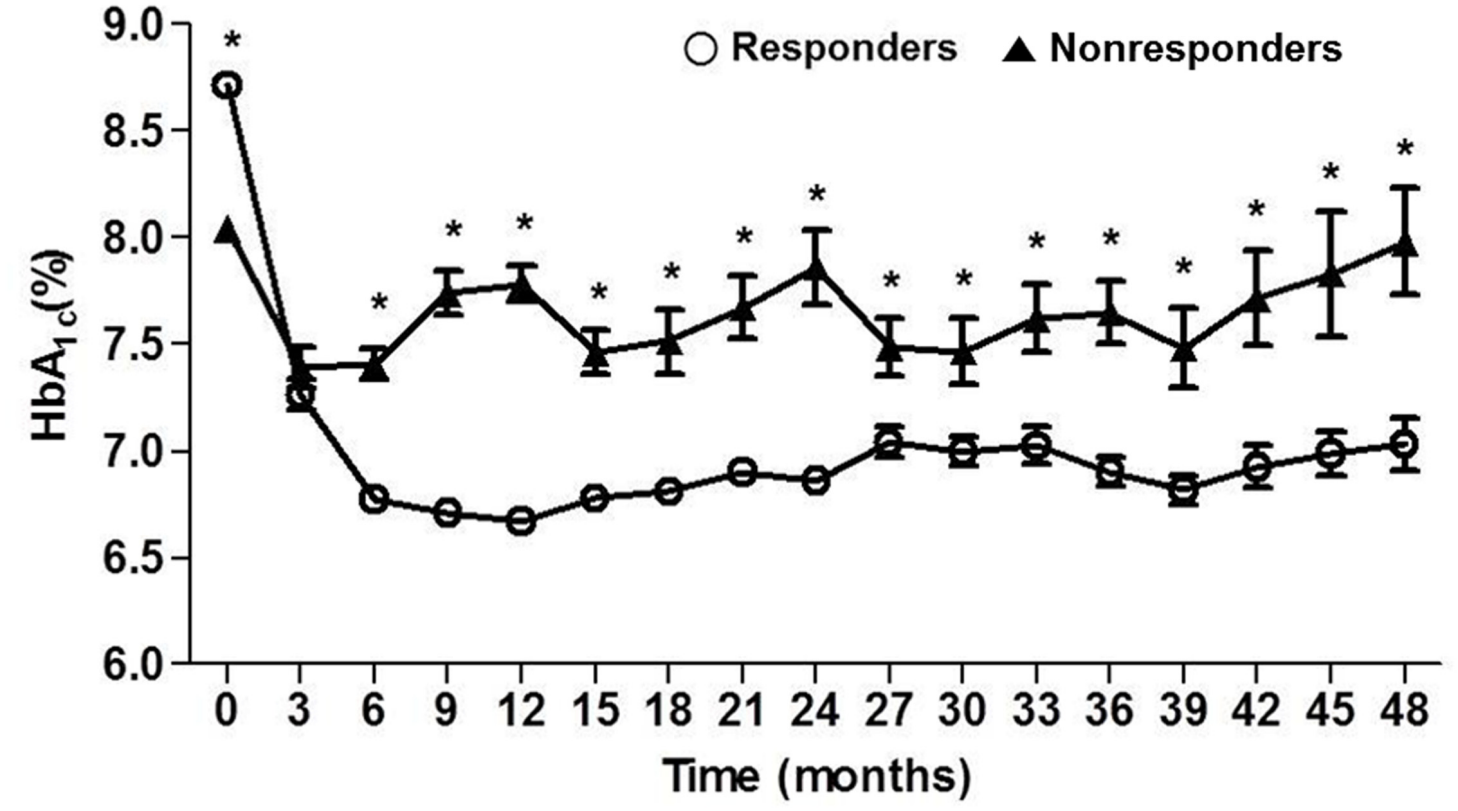
Comparison of HbA_1c_ levels for 48 months between responders (n = 315) and nonresponders (n = 42) when response was defined as ≥0.8% of HbA_1c_ reduction from baseline or attainment of target HbA_1c_ (≤7.0%) at the end of 4 years’ follow-up. * *P* < 0.001 for responder vs. nonresponder group.

When the HbA_1c_ levels for the responder and nonresponder groups were compared over the 4-year study period (Fig 2), HbA_1c_ levels for the responders decreased by 1.57 ± 1.13% during the first 3 months of treatment, decreased by a further 0.53 ± 0.96% during the next 3 months, and then were maintained at a relatively constant level to the 4-year follow-up.

In contrast, the mean HbA_1c_ level in the nonresponders decreased by 0.6% from the baseline during the first 3 months but fluctuated at levels around 7.5% to 8.0% after that time. During the 4 years of the study, the mean difference of HbA_1c_ between the responder and nonresponder groups was 0.73% (*P*<0.001).

**Fig 3:**
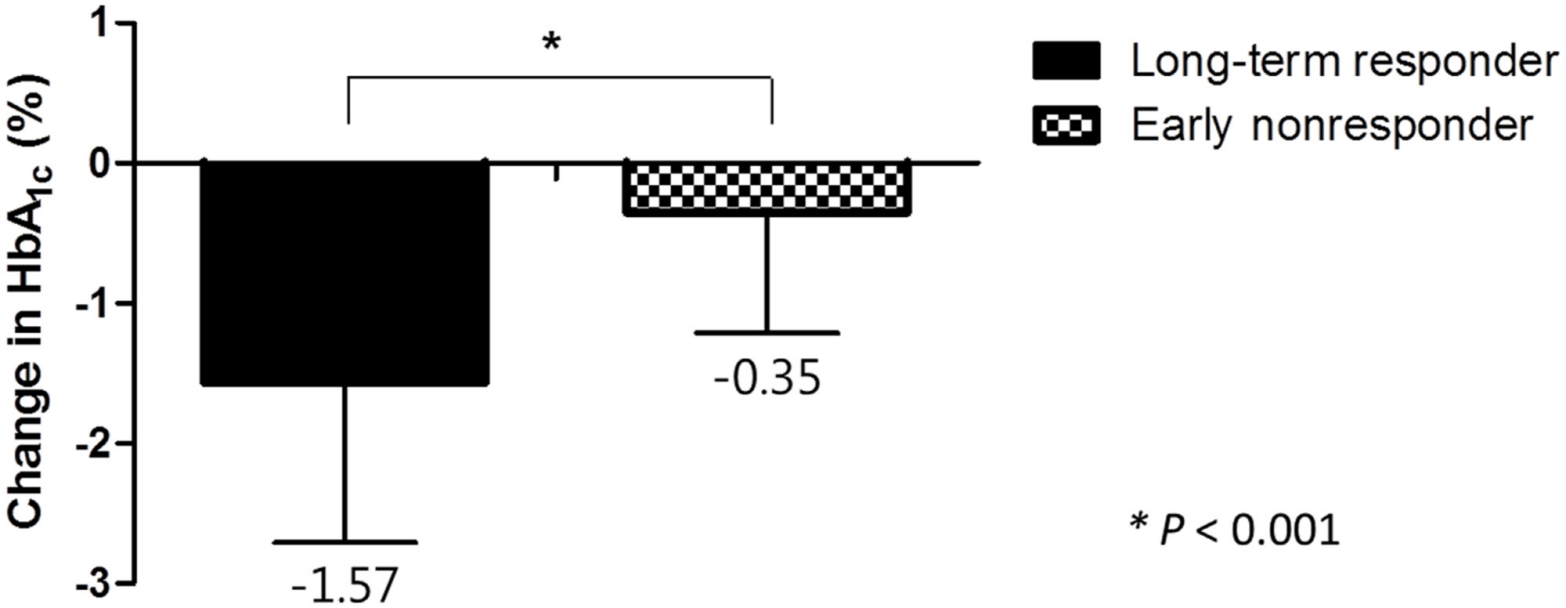
Reduction in HbA_1c_ (%) after 3 months in long-term responders and early nonresponders.

When the HbA_1c_ levels of long-term responders were compared with those of early nonresponders (those who failed to respond at the 1-yearevaluation), the HbA_1c_ levels decreased by 1.57±1.10% and 0.35±0.90% in the long-term responders and early nonresponders, respectively (*P*<0.001) (Fig 3). The change of HbA_1c_ levels from the baseline to the last follow-up in the long-term responders was also greater than that in the early nonresponders (−2.0±1.2% vs. −0.1±0.8%, *P*<0.001).

The most common antidiabetic agent added for rescue was sulfonylurea (92.6%). The other agents used to achieve the therapeutic glycemic goal were insulin (5.9%), thiazolidinedione (0.9%), and meglitinide (0.9%).

### Predictive factors for long-term response to initial combination treatment with sitagliptin and metformin

Multiple regression analyses were conducted to identify factors that could predict the long-term response to initial combination treatment with sitagliptin and metformin for up to 4 years (Table 2). A shorter duration of diabetes before treatment was an independent predictor for a greater reduction of HbA_1c_ in models 1–3. In model 3, the low HOMA-β and high HOMA-IR at the baseline were significant independent predictive factors for a greater reduction of HbA_1c_ (both *P*<0.001). No family history of diabetes was also a predictor of long-term response in model 3. When all of the confounders were included in the multivariable regression analysis in model 4, only a high HbA_1c_ level at baseline was found to be a predictive factor (*P*<0.001).

**Table 2:** The predictive factors for long-term HbA_1c_ reduction of initial combination therapy with sitagliptin and metformin.

	Model 1		Model 2		Model 3		Model 4	
	β	*P*	β	*P*	β	*P*	β	*P*
Age (years)	**−0.018**	0.038	**0.026**	0.012	−0.013	0.164	−0.001	0.873
Sex (1 = male, 2 = female)	**−**0.049	0.843	−0.135	0.626	−0.029	0.903	−0.161	0.298
SBP(mmHg)	−0.001	0.886	−0.002	0.763	−0.002	0.734	0.004	0.368
BMI (kg/m2)	−0.005	0.840	0.005	0.872	−0.020	0.497	−0.019	0.301
Duration of diabetes (years)	**−0.050**	0.014	**−0.073**	0.003	**−0.064**	0.002	−0.023	0.095
Family history of diabetes	−0.277	0.138	−0.406	0.052	**−0.469**	0.009	−0.199	0.090
Alcohol (1 = moderate, 2 = heavy)	−0.051	0.782	−0.027	0.894	−0.145	0.399	−0.060	0.594
Smoking (1 = never, 2 = current/ex-smoker)	−0.051	0.782	−0.197	0.175	−0.106	0.395	−0.098	0.226
Exercise (1 = irregular, 2 = regular)	−0.130	0.315	−0.154	0.198	−0.093	0.362	−0.014	0.837
Triglyceride (mg/dl)*			0.001	0.527	0.001	0.380	0.001	0.732
HDL-C (mg/dl)*			0.005	0.616	−0.001	0.952	0.001	0.966
ALT (IU/ml)*			−0.286	0.131	−0.278	0.081	−0.071	0.494
eGFR (ml/min/1.73m2)			**−**0.002	0.769	0.001	0.983	0.004	0.285
HOMA-β*					**0.172**	<0.001	0.010	0.685
HOMA-IR*					**−1.083**	<0.001	−0.150	0.205
Baseline HbA1c (%)							**0.857**	<0.001

SBP, systolic blood pressure; BMI, body mass index; HDL-C, high-density lipoprotein cholesterol; ALT, alanine aminotransferase; eGFR, estimated glomerular filtration rate.

* analyzed after log transformation.

Model 1: Included baseline age, sex, SBP, BMI, duration of diabetes, family history of diabetes, alcohol consumption, smoking habit, exercise

Model 2: Model 1 + triglyceride, HDL-C, ALT, eGFR

Model 3: Model 2 + HOMA-IR and HOMA-β

Model 4: Model 3 + baseline HbA_1c_

In the subgroup analysis based on the median HbA_1c_ value in the patients with a 4-year follow-up, a short duration of diabetes, no family history of diabetes, low HOMA-β, and high HbA_1c_ at baseline were significant predictors for reduction of HbA_1c_ in the patients with HbA_1c_ ≥8.5% at baseline after adjusting for all confounders (all *P*<0.05). In the patients with HbA_1c_ <8.5%, a high HbA_1c_ level at baseline was a significant predictor for reduction of HbA_1c_ with sitagliptin and metformin initial combination therapy.

**Fig 4:**
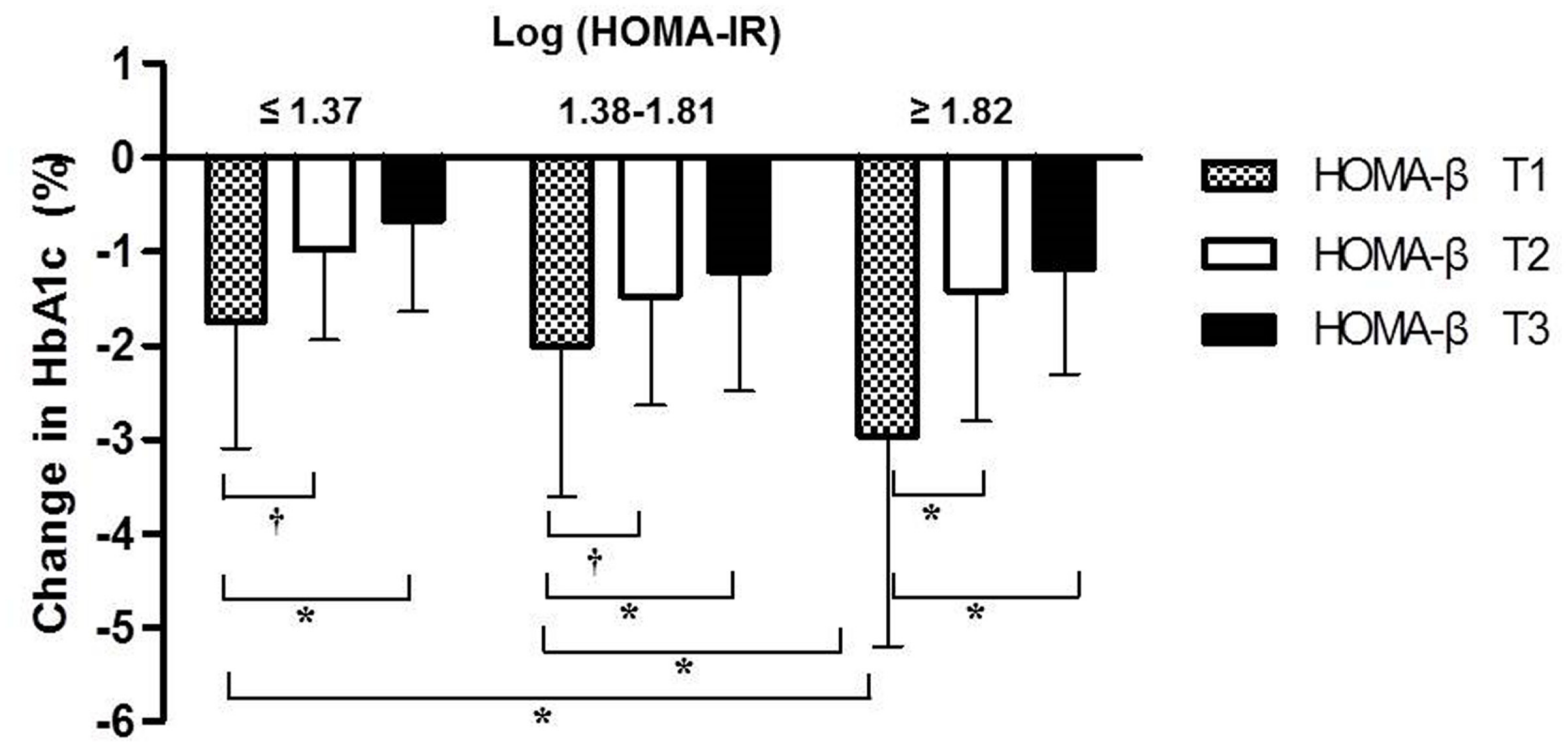
Changes in HbA_1c_ (%) after initial combination therapy with sitagliptin and metformin according to the tertiles (T) of HOMA-IR and HOMA-β at baseline. HOMA-IR and HOMA-β; homeostasis model assessment of insulin resistance and β-cell function. Logarithmically transformed values of HOMA-IR and HOMA-β were used for analyses. Log (HOMA-β) tertiles; T1 ≤3.65, 3.66 ≤T2 ≤4.17, T3 ≥4.18. * *P*<0.001, ^†^ *P*<0.05.

The changes observed in HbA_1c_ according to the baseline HOMA-IR and HOMA-β were presented in Fig 4. The reductions in HbA_1c_ were greater in the subgroups in the lower tertile of HOMA-β for any of the HOMA-IR tertiles (*P*<0.001). The patients in the lowest tertile of HOMA-β and the highest tertile of HOMA-IR showed the greatest reduction in HbA_1c_ level.

### Safety and tolerability

Initial combination therapy with sitagliptin and metformin was generally well tolerated, and there was no need for discontinuation of coadministration because of serious adverse experiences. No laboratory signs of hepatotoxicity or renal toxicity were encountered in any of the study subjects. Over the 4-year follow-up period, there were no cases of pancreatitis or pancreatic cancer. One patient discontinued the medication because of abdominal discomfort (bloating).

## Discussion

In this study conducted in a clinical practice setting, 72.2% of patients maintained are response to initial combination therapy with sitagliptin and metformin over a 4-year period, defined by a HbA_1c_ reduction ≥0.8% from baseline or attainment of the target HbA_1c_ (≤7.0%). Furthermore, the mean HbA_1c_ in the responder groups was stable throughout the 4-year period. The response rate increased to 88.2% in the fourth year after excluding patients who were lost to follow-up or were excluded for other reasons.

Recent studies have demonstrated the glycemic-lowering efficacy of sitagliptin therapy either in combination with other antidiabetic drugs or as monotherapy in patients with T2D [8–12]. However, the duration of these studies only ranged from 3 months to 2 years. Most recently, the ODYSSÉE observational study group reported the 3-year durability of combination therapy with sitagliptin and metformin [16]. This study showed that dual therapy with metformin combined with sitagliptin was more durable than metformin combined with sulfonylurea (median duration: 43.2 months vs. 20.2 months, *P*<0.001). The VERIFY study, which was designed to investigate the long-term durability of initial combination therapy with vildagliptin and metformin, commenced in 2013 [17]. Therefore, the present study is the longest study undertaken so far that has investigated the durability of initial combination therapy with a DPP-4 inhibitor and metformin.

In the present study, initial combination therapy with sitagliptin and metformin showed a significant reduction of the HbA_1c_ level by 1.5% in the first year, and its efficacy was maintained for 4 years. The good overall efficacy of this initial combination therapy demonstrated in this study is consistent with the results of previous studies [8,12,18–20]. Goldstein *et al*. reported that the mean reduction of HbA_1c_ was 1.4% for initial dual therapy of sitagliptin and metformin in drug-naïve patients with uncontrolled T2D (mean baseline HbA_1c_ = 8.8%) [18]. Another study with a sitagliptin add-on to metformin showed greater reductions of HbA_1c_, by 1.4% at week 30, compared with the placebo group [19].

In this study, 35.4% (315/890) of the patients remained as responders at the end of the fourth year. The A Diabetes Outcome Progression trial (ADOPT) showed that the proportion of patients achieving a target HbA_1c_ <7% at the 4-year follow-up were 40%, 36%, and 26% for rosiglitazone, metformin and glyburide, respectively [21]. Another study with nateglinide and gliclazide in combination with metformin showed that the proportions of patients achieving HbA_1c_ <7% were 40.0% and 47.4% at the end of 1year, respectively [22]. Thus, an absolute response rate of 35.4% at the end of the fourth year is comparable with other studies.

Based on the number of subjects enrolled in the previous year, more than 70% of patients responded to initial combination treatment with sitagliptin and metformin for 4 years, with the response rate increasing to 88.2% in the fourth year. Thus, the response rate increased over time. However, the response rate might be underestimated because some patients were excluded from follow-up for receiving additional antidiabetic agents or replacing sitagliptin with other antidiabetic agents, even though their HbA_1c_ levels decreased by ≥0.8% following initial combination therapy. Nonresponding patients and those who were lost to follow-up were also excluded from the calculation of the overall response rate. Thus, the present study reflects the pattern seen in clinical practice, which most clinical studies cannot replicate.

It is well known that a high HbA_1c_ level prior to treatment is a strong predictive factor of the glucose-lowering response of any kind of antidiabetic drug [23–25]. In this study, a high baseline HbA_1c_ level was the most significant factor in the response to long-term treatment with coadministration of sitagliptin and metformin. In the subgroup analysis of patients with HbA_1c_ levels ≥8.5% at baseline, a low HOMA-β at baseline was an independent predictor of a better response for individuals commencing combination therapy with sitagliptin and metformin. This finding is consistent with the results of our previous study [26]. In contrast, there were no significant predictive factors in the low-baseline-HbA_1c_ group. The lower reduction of HbA_1c_ might be a reason for this. In the present study, the early nonresponders had lower levels of regular exercise, a longer duration of diabetes, lower baseline HbA_1c_ levels, higher triglyceride levels, lower HDL-C levels, and higher HOMA-β than the long-term responders.

There is evidence indicating that DPP4 inhibitors increase β-cell function in humans [27]. Our finding that the reduction in HbA_1c_ was greater in patients with low β-cell function at any level of insulin resistance (assessed by HOMA) also suggests that sitagliptin showed long-term glucose-lowering efficacy by increasing β-cell function. However, little is known of the long-term direct effects of DPP-4 inhibitors on β-cell mass in humans.

In the current study, initial combination therapy with sitagliptin and metformin was suggested for the drug-naïve patients with T2D. Many of the recent studies present the efficacy and safety of the initial dual therapy in subjects with new-onset T2D, particularly those subjects with HbA_1c_ ≥7.5% [8,28,29]. In a 104-week study, initial combination therapy with sitagliptin and metformin provided substantial glycemic improvements and were well tolerated in patients with T2D and inadequate glycemic control (HbA_1c_ = 7.5–11%), despite following good dietary and exercise guidelines, were compared [8]. In a 26-week study investigating the efficacy and safety of the dipeptidyl peptidase-4 inhibitor alogliptin plus metformin initial combination therapy, the combination therapy compared favorable to monotherapy of either drug in drug-naïve T2D patients (HbA_1c_ = 7.5–10.0%). Alogliptin plus metformin initial combination therapy was well tolerated and more efficacious in controlling hyperglycemia in these patients than monotherapy of either drug [29]. In addition, the Korean Diabetes Association treatment guideline allows physicians to prescribe initial dual combination for diabetic patients who have HbA_1c_ ≥7.5% on their first visit [30] (S1 Fig).

During follow-up, another antidiabetic agent was added or initial combination of sitagliptin and metformin was changed for the treatment of patients at the physician’s discretion to achieve the target HbA_1c_ goal (≤7.0%) according to the American Diabetes Association (ADA) and KDA guidelines [30,31], even though their HbA_1c_ levels had decreased by ≥0.8% following initial combination therapy.

In this study, the mean HbA_1c_ level of nonresponders was 7.5‒8.0%, which was higher than recommended [30,31]. In clinical practice, it is practically impossible to ensure that all patients with diabetes achieve the target HbA_1c_ levels. It has been reported that only about one-third of diabetic patients reach target HbA_1c_ levels around the world [32–34]. In addition, higher HbA_1c_ levels are allowed for older patients or patients with comorbidities to avoid hypoglycemia.

In the current study, we used two definitions for responders: reduction in HbA_1c_ levels by 0.8% from baseline and attainment of the target HbA_1c_ level (≤7.0%). These two groups may have different characteristics. Among responders, 81.3% (n = 256) fulfilled both definitions (Group A); only 4.1% (n = 13) belonged to the target HbA_1c_ ≤7.0% (Group B) and 14.6% (n = 46) belonged to the reduction in HbA_1c_ ≥0.8% (Group C), exclusively. When Group A+B and Group A+C were compared, there was no difference between the variables in the regression models, indicating the homogeneity of the long-term responders in our study (S1 and S2 Tables).

This study has several strengths. First, to the best of our knowledge, this is the first study that has investigated the 4-year efficacy of initial combination therapy with sitagliptin and metformin. Second, the results of the present study were obtained in a clinical practice setting. Third, physicians interviewed all of the patients, particularly in relation to their other medical history, which might be related to the efficacy, reporting of possible adverse events, and maintaining drug compliance at each visit.

There are also some limitations in the current study. First, there was no active comparator in this study. Second, the gold standard methods for evaluating pancreatic β-cell function and insulin resistance, such as an insulin clamp study, were not applied. Third, the choice of ≥0.8% of HbA_1c_ reduction as a threshold of efficacy is arbitrary, although several clinical studies with metformin and DPP-4 inhibitors combination have shown average reduction of HbA_1c_ by ≥0.8% [1,3,5,7,35]. Fourths, changes resulting from the use of other medications, such as statins or renin—angiotensin system blockers, may have affected the glucose metabolism. Last, the annual lost to follow-up rate in this study was 10.6‒23.1%, which seems to be slightly high. However, recent clinical studies with initial combination therapy including DPP-4 inhibitors [8,28,32] showed similar lost to follow-up rate (19.6‒32%). The follow-up rate is generally higher in controlled drug trials than observational or real world studies. For example, in a real world study using the Hungarian National Health Insurance data, the first-year persistence rate with sulfonylurea and metformin was 55.8% [36]. In a study using a diabetes registry, the first-year follow-up rate in the DPP-4 inhibitors + metformin group was 73.7% [37]. In another observational study, the 6-month follow-up rate for patients receiving sitagliptin and metformin combination therapy was 71.7% [38]. Thus, the follow-up rate in this study is comparable with those of other studies and is unlikely to change the main finding. However, there is still a possibility that this numerically low follow-up rate may attenuate the study results.

In conclusion, the current study suggests that initial combination therapy with sitagliptin and metformin is an attractive therapeutic strategy with long-term durability and a good safety profile for patients with uncontrolled T2D.

## Supporting Information

S1 Fig Korean Diabetes Association Treatment Guideline for Diabetes Mellitus. (DOCX)

S1 Table The predictive factors for long-term HbA_1c_ reduction of initial combination therapy with sitagliptin and metformin in patients with attainment of the target HbA_1c_ (≤7.0%). (DOCX)

S2 Table The predictive factors for long-term HbA_1c_ reduction of initial combination therapy with sitagliptin and metformin in patients with HbA_1c_ reduction ≥0.8% from the baseline. (DOCX)
