# Supplementary figures and images for: Four-Year Durability of Initial Combination Therapy with Sitagliptin and Metformin in Patients with Type 2 Diabetes in Clinical Practice; COSMIC Study
Source: PLoS One. 2015 Jun 12;10(6):e0129477. doi: 10.1371/journal.pone.0129477 (PMC4466580; doi:10.1371/journal.pone.0129477)

**S1 Fig. Korean Diabetes Association Treatment Guideline for Diabetes Mellitus**


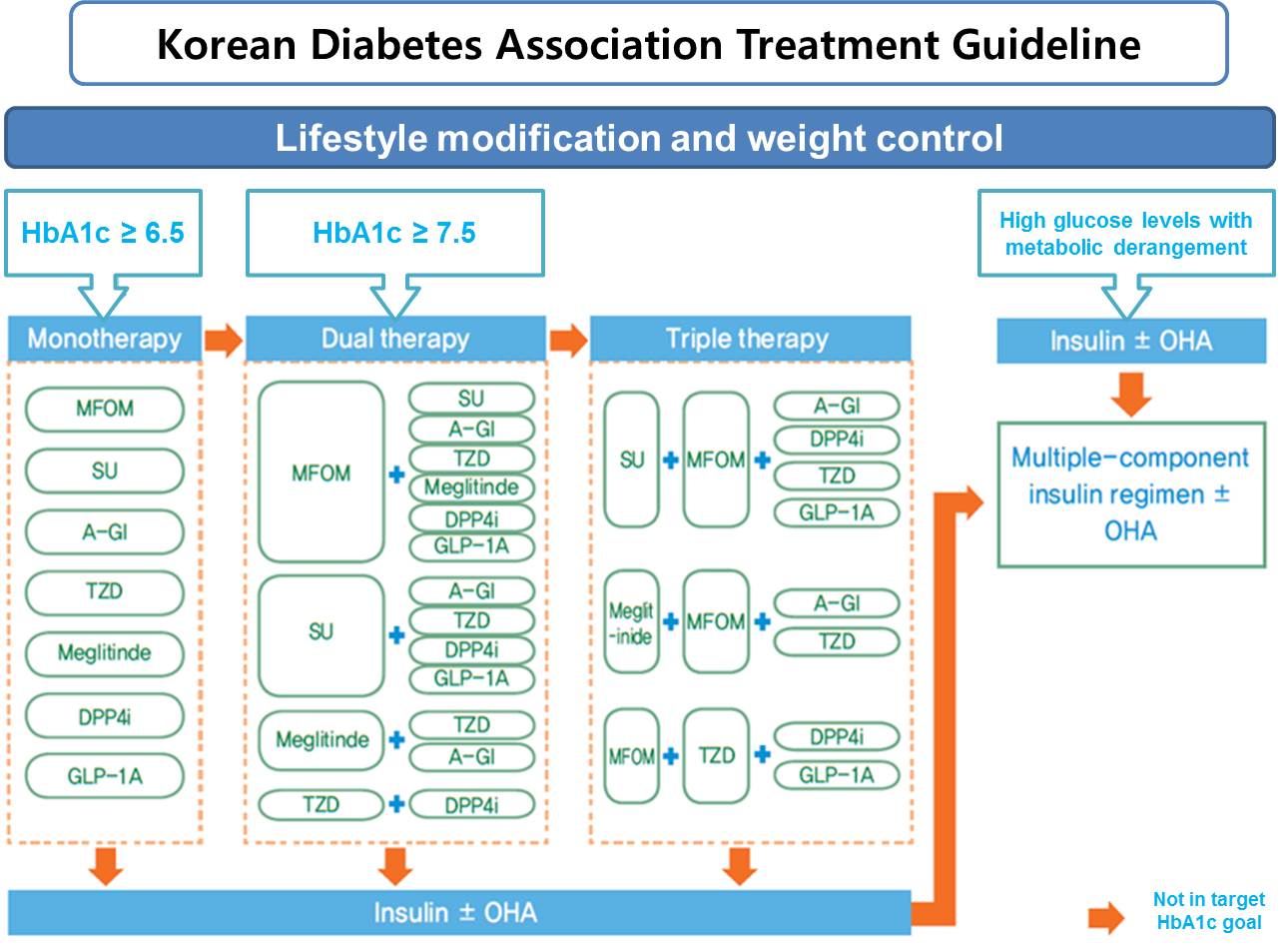

Supplement: S1 Fig — (DOCX) [file pone.0129477.s001.docx]
