# Supplementary material for: Four-Year Durability of Initial Combination Therapy with Sitagliptin and Metformin in Patients with Type 2 Diabetes in Clinical Practice; COSMIC Study
Source: PLoS One. 2015 Jun 12;10(6):e0129477. doi: 10.1371/journal.pone.0129477 (PMC4466580; doi:10.1371/journal.pone.0129477)
Supplement: S1 Table — (DOCX) [file pone.0129477.s002.docx]

| **S1 Table. The predictive factors for long-term HbA_1c_ reduction of initial combination therapy with sitagliptin and metforminin patients with attainment of the target HbA1c (≤7.0%)** | | | | | | | | |
| --- | --- | --- | --- | --- | --- | --- | --- | --- |
|  | Model 1 | | Model 2 | | Model 3 | | Model 4 | |
|  | β | *P* | β | *P* | β | *P* | β | *P* |
| Age (years) | -0.017 | 0.066 | **-0.022** | 0.039 | -0.009 | 0.322 | -0.001 | 0.790 |
| Sex (1 = male, 2 = female) | 0.127 | 0.648 | 0.220 | 0.492 | 0.333 | 0.214 | 0.021 | 0.848 |
| SBP(mmHg) | -0.003 | 0.656 | 0.001 | 0.958 | 0.002 | 0.780 | 0.002 | 0.380 |
| BMI (kg/m^2^) | -0.006 | 0.863 | -0.022 | 0.575 | 0.006 | 0.858 | 0.016 | 0.257 |
| Duration of diabetes (years) | **-0.055** | 0.013 | **-0.064** | 0.020 | **-0.052** | 0.016 | -0.002 | 0.804 |
| Family history of diabetes | -0.304 | 0.141 | -0.428 | 0.073 | **-0.459** | 0.018 | -0.055 | 0.501 |
| Alcohol (1 =moderate, 2 =heavy) | 0.360 | 0.088 | 0.363 | 0.127 | 0.030 | 0.875 | -0.030 | 0.711 |
| Smoking (1=never, 2=current/ex-smoker) | -0.235 | 0.123 | -0.159 | 0.360 | 0.042 | 0.771 | 0.097 | 0.103 |
| Exercise (1 = irregular, 2 = regular) | -0.184 | 0.135 | -0.235 | 0.091 | -0.074 | 0.507 | -0.015 | 0.752 |
| Triglyceride (mg/dl)* |  |  | 0.001 | 0.623 | 0.001 | 0.978 | 0.001 | 0.441 |
| HDL-C (mg/dl)* |  |  | 0.001 | 0.978 | -0.004 | 0.660 | -0.004 | 0.280 |
| ALT (IU/ml)* |  |  | -0.095 | 0.660 | -0.155 | 0.366 | -0.005 | 0.948 |
| eGFR (ml/min/1.73m^2^) |  |  | -0.009 | 0.222 | -0.006 | 0.328 | 0.001 | 0.568 |
| HOMA-β* |  |  |  |  | **-1.176** | <0.001 | **-0.217** | 0.010 |
| HOMA-IR* |  |  |  |  | **0.181** | <0.001 | 0.009 | 0.604 |
| Baseline HbA_1c_ (%) |  |  |  |  |  |  | **0.913** | <0.001 |
| *analyzed after log transformation. Model 1: Included baseline age, sex, SBP, BMI, duration of diabetes, family history of diabetes, alcohol consumption, smoking history, exercise habits, Model 2: Model 1 + triglyceride, HDL-C, ALT, eGFR, Model 3: Model 2 + HOMA-IR and HOMA-β, Model 4: Model 3 + baseline HbA_1c_ | | | | | | | | |
