# Supplementary material for: Four-Year Durability of Initial Combination Therapy with Sitagliptin and Metformin in Patients with Type 2 Diabetes in Clinical Practice; COSMIC Study
Source: PLoS One. 2015 Jun 12;10(6):e0129477. doi: 10.1371/journal.pone.0129477 (PMC4466580; doi:10.1371/journal.pone.0129477)
Supplement: S2 Table — (DOCX) [file pone.0129477.s003.docx]

| **S2 Table. The predictive factors for long-term HbA_1c_ reduction of initial combination therapy with sitagliptin and metforminin patients with HbA1c reduction** ≥**0.8% from the baseline** | | | | | | | | |
| --- | --- | --- | --- | --- | --- | --- | --- | --- |
|  | Model 1 | | Model 2 | | Model 3 | | Model 4 | |
|  | β | *P* | β | *P* | β | *P* | β | *P* |
| Age (years) | **-0.021** | 0.016 | **-0.024** | 0.018 | -0.009 | 0.330 | 0.001 | 0.870 |
| Sex (1 = male, 2 = female) | -0.008 | 0.976 | -0.010 | 0.973 | 0.109 | 0.644 | -0.016 | 0.906 |
| SBP(mmHg) | -0.006 | 0.362 | -0.004 | 0.569 | -0.002 | 0.698 | 0.002 | 0.476 |
| BMI (kg/m^2^) | 0.033 | 0.240 | 0.031 | 0.326 | 0.010 | 0.723 | 0.004 | 0.783 |
| Duration of diabetes (years) | **-0.047** | 0.022 | **-0.065** | 0.008 | **-0.060** | 0.004 | -0.015 | 0.187 |
| Family history of diabetes | -0.229 | 0.228 | -0.324 | 0.130 | **-0.396** | 0.025 | -0.147 | 0.140 |
| Alcohol (1 =moderate, 2 =heavy) | 0.061 | 0.741 | 0.016 | 0.939 | -0.082 | 0.622 | -0.043 | 0.645 |
| Smoking (1=never, 2=current/ex-smoker) | -0.133 | 0.326 | -0.069 | 0.650 | 0.039 | 0.761 | 0.043 | 0.552 |
| Exercise (1 = irregular, 2 = regular) | -0.155 | 0.157 | -0.165 | 0.175 | -0.117 | 0.243 | -0.033 | 0.555 |
| Triglyceride (mg/dl)* |  |  | 0.001 | 0.965 | 0.001 | 0.899 | 0.001 | 0.662 |
| HDL-C (mg/dl)* |  |  | 0.003 | 0.782 | -0.007 | 0.448 | -0.002 | 0.704 |
| ALT (IU/ml)* |  |  | -0.276 | 0.157 | -0.314 | 0.460 | -0.086 | 0.333 |
| eGFR (ml/min/1.73m^2^) |  |  | -0.002 | 0.713 | -0.002 | 0.785 | 0.002 | 0.579 |
| HOMA-β* |  |  |  |  | **-1.069** | <0.001 | -0.115 | 0.255 |
| HOMA-IR* |  |  |  |  | **0.158** | <0.001 | 0.002 | 0.937 |
| Baseline HbA_1c_ (%) |  |  |  |  |  |  | **0.837** | <0.001 |
| *analyzed after log transformation. Model 1: Included baseline age, sex, SBP, BMI, duration of diabetes, family history of diabetes, alcohol consumption, smoking history, exercise habits, Model 2: Model 1 + triglyceride, HDL-C, ALT, eGFR, Model 3: Model 2 + HOMA-IR and HOMA-β, Model 4: Model 3 + baseline HbA_1c_ | | | | | | | | |
